# Vaccination as a social practice: towards a definition of personal, community, population, and organizational vaccine literacy

**DOI:** 10.1186/s12889-023-16437-6

**Published:** 2023-08-08

**Authors:** Chiara Lorini, Marco Del Riccio, Patrizio Zanobini, Roberto Luigi Biasio, Paolo Bonanni, Duccio Giorgetti, Valerio Allodola Ferro, Andrea Guazzini, Olfa Maghrebi, Vieri Lastrucci, Lisa Rigon, Orkan Okan, Kristine Sørensen, Guglielmo Bonaccorsi

**Affiliations:** 1 Department of Health Sciences, University of Florence, Viale Giovanni Battista Morgagni 48, 50134 Florence, Italy; 2 Health Literacy Laboratory (HeLiLab), University of Florence, Viale Giovanni Battista Morgagni 48, 50134 Florence, Italy; 3 Fondazione Giovanni Lorenzini, Viale Piave 35, 20129 Milan, Italy; 4 Medical School of Specialization in Hygiene and Preventive Medicine, University of Florence, Largo Giovanni Alessandro Brambilla 3, 50134 Florence, Italy; 5 Department of Law, Economics and Human Sciences, Mediterranea University of Reggio Calabria, Via Dell’Università 25, 89124 Reggio Calabria, Italy; 6 Department of Education, Literatures, Intercultural Studies, Languages and Psychology, University of Florence, Via Di San Salvi 12, 50135 Florence, Italy; 7 Epidemiology Unit, Meyer’s Children University Hospital, Viale Gaetano Pieraccini, 24, 50139 Florence, Italy; 8 Department of Sport and Health Sciences, Technical University of Munich, 80992 Munich, Germany; 9 Global Health Literacy Academy, Viengevej 100, 8240 Risskov, Denmark

**Keywords:** Vaccine literacy, Vaccine hesitancy, Organization, Health literacy

## Abstract

**Background:**

A comprehensive and agreed-upon definition of vaccine literacy (VL) could support the understanding of vaccination and help policy-makers and individuals make informed decisions about vaccines.

**Methods:**

To shed some light on this debate and provide clarity, a scoping review was conducted to collect, summarize, and analyse available definitions of VL. Based on the findings of the scoping review, a new and comprehensive definition was proposed by a panel of experts.

**Results:**

Fifty-three articles were included, and two of them appeared to be the milestones around which the other definitions were grouped. The new definition proposed by the panel of experts included not only the personal perspective, but also the community, population, and organizational perspectives. Moreover, due to the increasing complexity of the social context with respect to the ability to navigate, understand, and use information and services, the definition of organizational vaccine literacy and the attributes of a vaccine literate healthcare organization have been proposed.

**Conclusion:**

The new definition can contribute to the overall paradigm of health literacy and its distinct component of vaccine literacy, possibly improving the implementation of public health strategies to allow vaccination to be understood as a social practice by the entire community. This study describes the conceptual foundations, the competencies, and the civic orientation to be considered when developing measurement tools devoted to assessing VL at the different levels and in different contexts.

**Supplementary Information:**

The online version contains supplementary material available at 10.1186/s12889-023-16437-6.

## Introduction

The COVID-19 pandemic has had enormous repercussions on health and health care systems worldwide in terms of mortality, morbidity, and economic costs. Lockdowns and other nonpharmaceutical interventions (NPIs) adopted by governments in the early stages of the pandemic were followed by COVID-19 vaccination campaigns and other interventions to promote vaccination and increase coverage rates (e.g., the EU Digital COVID Certificate) [1]. The need for a global mass vaccination created – as a sort of retaliation – the premises for anti-vaccine movements and anti-vaccine demonstrations by those who opposed mandatory COVID-19 vaccination. This opposition to vaccination is relevant for understanding the landscape of vaccine hesitancy. Notably, even health care professionals, who have generally received training about vaccines and vaccination strategies, have been observed among those expressing hesitancy towards the COVID-19 vaccine [2, 3]. This observation underscores the complexity of vaccine hesitancy and warrants further exploration. Moreover, it is crucial to recognize that individuals who refuse the COVID-19 vaccine may not necessarily oppose vaccination in general but rather harbour concerns specific to this vaccine [4]. To explore this widespread aversion to vaccination or specific vaccines, it is important to examine studies on how to increase the willingness to be vaccinated against COVID-19 and people’s and communities’ awareness of the real value of COVID-19 vaccination [5, 6].

In this scenario, health literacy (HL) has received an increasing attention [7, 8]. In particular, HL and vaccine literacy (VL) can help people make decision about vaccination [9]. The concept of HL has been widely explored in the literature and can be defined as the knowledge, motivation, and competencies to access, understand, appraise and apply information to form judgements and make decisions regarding health care, disease prevention and health promotion to meet health demands [10, 11]. However, VL is still a matter of debate. For instance, although the term VL is frequently used to refer to the abilities that may shape intentions to receive vaccination and vaccine uptake, there is a lack of consensus on the definition of VL, that is, whether it can be considered a specific part of HL applied to vaccines and vaccination or whether it involves distinct meanings, knowledge and skills and whether it should refer only to personal knowledge and abilities or should also include organizational and population aspects [12]. In fact, as for HL, in addition to the personal level, also the community and organizational levels have been described. In particular, a health-literate community is able to gather information on social determinants of health, to mobilize the collective resources to act upon these determinants, and to advocate efficiently for structural changes in order to improve the daily living conditions of its members [13]. From this perspective, the HL of the community is fundamental for its own empowerment, which implies community ownership and actions that explicitly aim from social and political change. Organizational health literacy (OHL) is the degree to which organizations equitably enable individuals to find, understand, and use information and services to inform health-related decisions and actions for themselves and others [14]. In particular, when considering OHL, the focus is on the organization-wide effort made by different entities (e.g. health care organizations, policy-makers, the communications system, schools) to make it easier for people to navigate, understand, and use information and services to take care of their health. When OHL is taken into account, culture and leadership, systems, policies and practices, and the workforce allow for the provision of services, programmes and information in ways that promote equitable access and engagement, that meet the diverse HL needs and preferences of all people, and that support individuals and communities to participate in decisions regarding their health and well-being (health literacy responsiveness) [15].

To date, the relationship between HL and VL on the one hand, and vaccine hesitancy or acceptance on the other, remains largely unexplored. Studies upon it are still inconsistent, and the association varies according to population groups, vaccines, geographical areas, and measures of HL and VL used [16, 17]. Furthermore, to the best of our knowledge, no studies have shown a clear association between HL, VL, and confidence in vaccination in different health care contexts. The absence of a clear and widely adopted definition of VL may impede researchers and policy-makers from conducting effective research and developing interventions aimed at promoting understanding of vaccination. Therefore, the aim of this study is to propose a new definition of VL.

## Methods

The methodology used for proposing a new definition of VL can be divided into two main steps**.** First, a scoping review was conducted to collect, summarize, and analyse all the VL definitions. Second, based on the findings of the scoping review, a panel of experts proposed a comprehensive definition that encompassed community, population, and organizational perspectives to provide stakeholders with a fresh paradigm to implement public health strategies.

The literature review was conducted according to Peters et al.’s methodology and the PRISMA guidelines to perform a systematic scoping review [18, 19]. Six steps were followed: identification of the research questions; search of relevant studies from different databases; study selection according to predefined eligibility criteria; data extraction; analysis of the findings; and discussion of the implications for policies, practice, and research.

### Search strategy and selection criteria

PubMed/MEDLINE, Embase, Web of Science, and Google Scholar were searched from inception to 1st December 2022 for original articles or abstracts (in case of conference proceedings) that presented a specific definition of “vaccine literacy”, disregarding the role played by the definition itself in the manuscript and its position in it (the definition could be placed in the introduction, in the methods, in the results, in the discussion, or in the conclusions, indifferently). The following search string was used: “vaccination literacy” OR “vaccin* literacy” OR “vaccin* health literacy” OR “vaccination health literacy”. Given that the aim of this scoping review was to find and discuss every specific definition of “vaccine literacy”, no articles in which the the two terms (“vaccine” and “literacy”) appeared separately were included. All identified citations were collated and uploaded into Endnote (Thomson Reuters, New York, NY, USA), and duplicates were removed. No time or geographic restrictions were applied; only full texts (or abstracts, in the case of conference proceedings) that were available in English were considered for inclusion.

The literature search and article selection were conducted independently by four researchers (MDR, DG, LR, VFA) and any disagreement was resolved by consensus or by a senior researcher (CL). This scoping review ultimately included all original abstracts or full papers that presented any definition of VL. The reference list of all eligible papers was inspected by means of backwards citation chaining to find additional articles that could be included.

Data extraction was performed using an internally piloted spreadsheet. We extracted the following information from each included article: author, country, and year in which the study was conducted; definition of VL; and references cited when defining VL.

### Data synthesis and expert panel

After scanning the eligible literature and extracting and coding the definitions, a content analysis was performed using the same approach that was applied by Sørensen et al. [10]. The core research team (University of Florence) discussed the analysis internally. Then, the results and a first draft of the definition were discussed with a panel of experts to reach a comprehensive definition of VL that could capture all the meanings and dimensions retrieved from the literature.

The core research team from the University of Florence consisted of a highly interdisciplinary group. It included three public health experts, two experts in vaccines and vaccinations strategies, two experts in health literacy and public health, two experts in psychological determinants of vaccine hesitancy, and one in pedagogy. The panel of experts comprised four representatives from the core research team, two prominent figures from European research groups working on HL with experience in logical framework building, a public health expert with extensive experience in HL associated with one of the most renowned children's hospitals in Europe, and an expert in vaccinology and in developing measurement tools for assessing VL. Two rounds of consultations—led by the principal investigator of the study—were performed to discuss the proposal developed by the core research group.

### Role of the founding source

The study was conducted without any sponsors or specific funding sources.

## Results

**Fig. 1:**
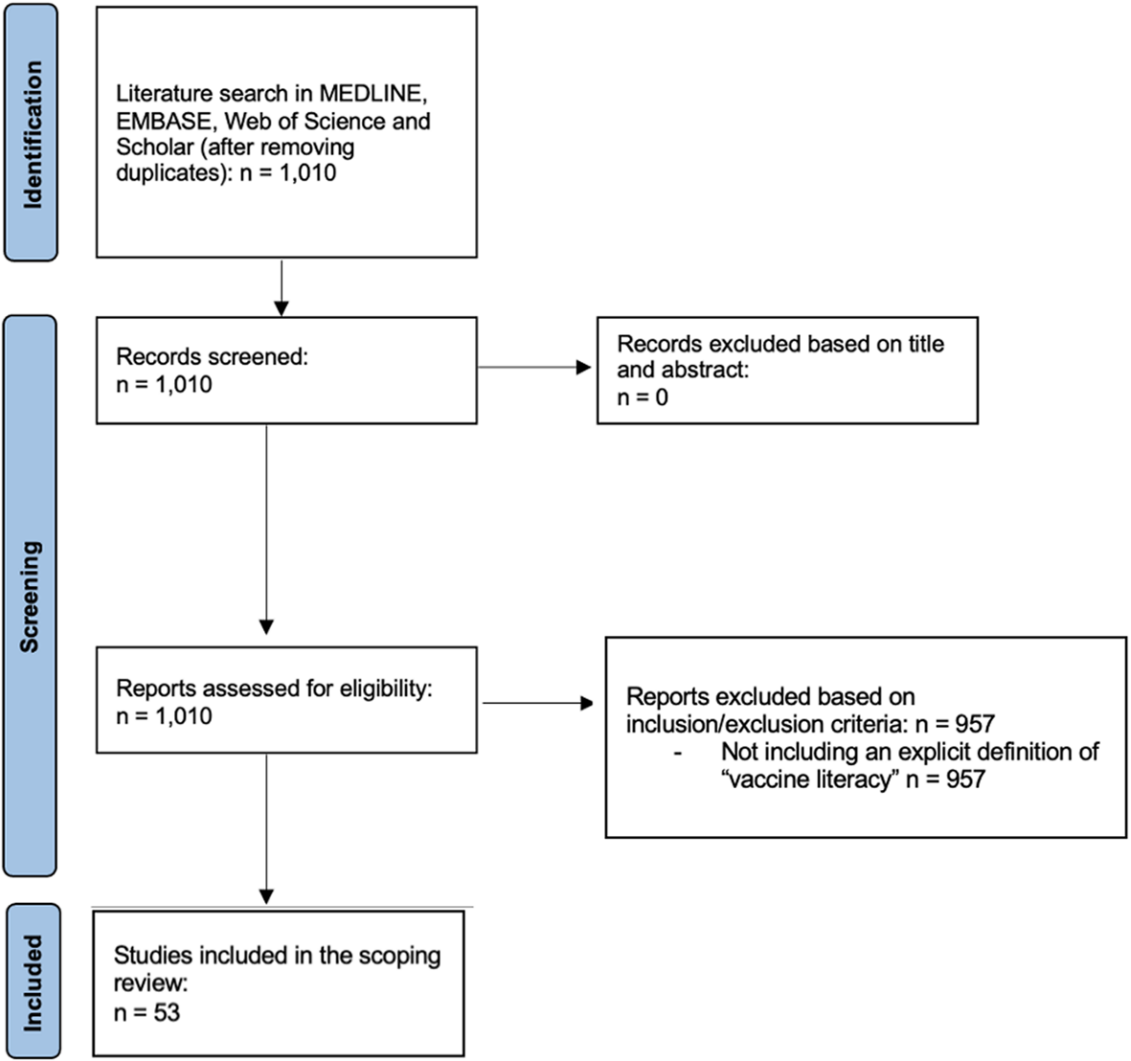
Selection process and results flow diagram

The literature search produced 1,010 nonduplicate entries. No additional articles were found by backwards citation chaining (Fig. 1). Considering the importance of not excluding any paper that presented a definition of VL, all 1,010 citations were read in full text (or abstract, in the case of conference proceedings). A total of 957 studies were excluded based on the inclusion criteria. Finally, 53 studies were found to be eligible and included in the scoping review [12, 16, 20–69].

### Scoping review: definitions of vaccine literacy

The 53 articles were published between 2009 and 2022 by groups from different countries. The US and Italy were the most represented countries (Supplementary Table 1). The 53 definitions reported in the included articles differed in terms of the references used as background and the areas/dimensions developed within the definition. In particular, two manuscripts appeared to be the milestones around which the other definitions were grouped. The definition of HL provided by Sørensen and colleagues [10], which involved the ability to access, understand, appraise and apply health information, integrated with the “functional”, “communicative”, and “critical”, that are the dimensions of health literacy introduced by Nutbeam et al. [70], was used several times as a main reference (Supplementary Table 1). Likewise, the definition reported by Ratzan et al. [62] involving “[…] not simply knowledge about vaccines, but also […] a system with decreased complexity to communicate and offer vaccines […]” was widely cited to include an organizational perspective in addition to a personal one. These two definitions identify two levels of VL: the personal VL, which entails individual abilities, and the organizational VL, which refers to the different degrees of complexity that an organization (or a system) that focuses on vaccine communication and offer may present. Along with these two dimensions, some authors identify a third, broader, dimension referred to as the population. Costantini and colleagues state that “VL is contingent on personal circumstances as well as the broader societal context, thus contributing to shape intentions to vaccinate and ultimately vaccine uptake” [36], while Budiyanti and colleagues report that “VL is a balance between individual, community and also population skills in complexity system” [31]. In another paper, Ratzan and colleagues also note that “VL occurs when the skills and abilities of people align with the content, processes, and systems needed to access and get vaccinated” [63], which is also highlighted by Masiello and colleagues [55].

As a result of the content analysis of the existing definitions, four clusters were identified that represented competencies, actions, objectives, and types of information reported in the different definitions with regards to personal, community/population or organization levels. Within each cluster, different terms and notions were identified to capture the essence of the different definitions. The results of the content analysis are summarized in Table 1.

**Table 1:** Summary of the results emerged of the content analysis

Levels	Clusters			
	**Competences/skills/abilities**	**Actions**	**Objectives**	**Information/issues/WHAT**
**Personal**	abilities knowledge skills motivation competence basic comprehension and interactive-critical literacy skills capabilities critical skills evaluation skills education capacity	to obtain to process to understand to collect to use to find to process data to process experience to critically analyse to apply meaning to understand to acquire to seek out to collect	to make informed vaccination decision to make decisions about vaccination to improve quality of life to improve critical system to improve systemic thinking to decide whether to accept the vaccination to promote health to maintain good health to facilitate the communication of messages to navigate the health system to be a critical consumer of anti-vaccine rhetoric to get vaccinated to act	health information information about vaccines information about immunization, disease prevention and health promotion vaccination programs the potential benefits of vaccination the risks of side effects the economic costs organizational process to access vaccination health issues treatment options the values of vaccination information services schedules and target diseases of the vaccines
**Community** **Population**	skills parents' knowledge public's skills public abilities	to find to judge to use to understand to read to comprehend to access to get to learn	to make decisions about children's and adults' vaccination to make informed immunization decisions for one’s children to achieve population vaccination to identify and prioritize the most essential information to appreciate the larger global impact of vaccines	vaccine-related information the credibility of information
**Organization**	a process	to develop a system to provide vaccine information	to decrease complexity to increase people's engagement with vaccines to communicate and offer vaccines	

### Results from the consultation of the expert panel

One of the outcomes of the expert panel consensus was to consider fundamental papers on HL to integrate preexisting definitions of VL [10, 11, 13, 71–73]. The main point of discussion focused on whether the definition of VL should include the vaccination uptake or acceptance as an outcome. Some argued that VL should concentrate solely on promoting informed decision-making regarding vaccinations. By the conclusion of the second consultation, a consensus was reached among all participants, agreeing not to include vaccination uptake or acceptance in the definition of VL.

As a result of both the content analysis of the included manuscripts and the two rounds of consultation of the expert panel, the following definitions of vaccine literacy, organizational vaccine literacy, and the description of the attributions of an vaccine literate healthcare organization were developed.

*Vaccine literacy is linked to health literacy. It entails people’s and communities’ knowledge, motivation, and competencies to access, understand, and critically appraise and apply information about immunization, vaccines, vaccination programmes, and organizational processes to access vaccination and to navigate the health system, in order to make informed decisions about vaccines for themselves, the members of their family, and the community, and to appreciate the larger global impact of vaccines with respect to population health. A vaccine literate community is able to mobilize collective resources, and to advocate for structural changes to make it easier to access vaccination.*

VL is a relational concept: it is the balance between personal, community and population skills, and the complexity/demand of the context. Within this perspective, the concept of organizational vaccine literacy (OVL) must be introduced.

*Organizational vaccine literacy is defined as an organizational effort (for example, definition of policies, resource allocations, consultations) to build an environment that supports individuals to navigate, understand, and use vaccine information and services to form judgements and make decisions for themselves, the members of their family, and their community*.

As for OHL, when OVL is taken into account, the different organizations that can influence the provision of vaccine information to individuals and communities adopt strategies that can promote equitable access and engagement, to meet different levels of VL skills, and to support individuals and communities in participating in the decision-making process regarding their choice to receive vaccination. As a result, all the settings of everyday life can be vaccine literate.

In this sense, a vaccine-literate environment (that is, the way information and services regarding vaccines and vaccinations are provided) can compensate for low individual, community, and population VL, and constitutes an opportunity to improve VL. Major stakeholders involved in developing a vaccine-literate environment are as follow:

- population;
- community;
- media and communicators;
- teachers, schools, and universities;
- policy-makers with responsibilities at local, regional, national, and global levels;
- civil society organizations and nongovernmental organizations;
- researchers;
- national immunization technical advisory groups, including scientific associations;
- patients’ associations;
- the private sector and employers;
- health care workers;
- health care organizations.

A vaccine-literate environment requires the development of effective partnerships between the involved actors and coordinated communication plans.

In particular, similarly to a health literate healthcare organization [71], a *vaccine literate health care organization* (VLHO) should:

1. inform people by clear, trustworthy, up-to-date evidence about vaccines and vaccination;
2. encourage questions and dialogue between people and health care workers about vaccines and vaccination;
3. communicate clearly the comparative risks and benefits of vaccination for each single person and the whole society;
4. develop a supportive environment that provides providing navigation assistance, and facilitate access to vaccination services to reduce structural or psychological barriers that make vaccination difficult (e.g. availability, affordability, accessibility, comprehensibility of information, effort, costs);
5. communicate clearly which individuals will have free access and which ones will have to pay to receive vaccines;
6. prepare its workforce to be vaccine-literate, and enhance communication skills;
7. include the served populations while designing, implementing and evaluating vaccine information materials and vaccination services;
8. meet the needs of populations with a range of VL skills while avoiding stigmatization;
9. design and distribute print, audiovisual and social media content that is easy to understand and act on;
10. have a leadership that provides (organizational) capacities, infrastructures and resources to ensure that the organization can be vaccine literate.

## Discussion

This study aimed to identify and synthesize concepts and definitions of VL, which were then reviewed and discussed by an expert panel. Consequently, a new comprehensive definition is proposed that encompasses the personal, community, population, and organizational perspectives of VL. In contrast to the previous definitions, this proposal introduces the community and population levels with respect to competencies (“*It entails people’s and communities’ knowledge, motivation, and competencies*) and the implications of being literate (“[…] *in order to make informed decision about vaccines for themselves, the members of their family, community")*. In particular, the inclusion of the appreciation of “*the larger global impact of vaccines with respect to the entire population health”* as a characteristic of vaccine literate individuals, communities, and populations, moves towards the civic orientation and engagement perspectives, which leads to community change (“*a vaccine-literate community is able to mobilize the collective resources, and to advocate for structural changes in order to make it easier to access vaccination”).* From this perspective, a vaccine-literate community plays a pivotal role in fostering a vaccine-literate environment. By actively addressing barriers and advocating for improved access to accurate and reliable information, a vaccine-literate community contributes positively to enhancing overall VL levels. This, in turn, helps empower individuals to make informed decisions about vaccinations and ultimately improves public health outcomes. These aspects are particularly relevant when considering preventive measures that can affect individual as well as the community and the society at large, as in the case of vaccine-preventable diseases. From this perspective, VL is strongly related to public HL [74, 75]. At the same time, this aspect is specific to VL as compared to HL since the decision to be vaccinated or not (usually) affects not only the individual but also the population at large. In fact, vaccinations serve as a vital strategy not only for safeguarding individuals and for achieving herd immunity as well as controlling, eradicating, and eliminating infectious diseases.

Additionally, considering the growing complexity of the social context in terms of navigating, comprehending, and utilizing information and services, we proposed the definitions of OVL and the attributes that describe a VLHO. These definitions serve as a foundation for implementing public health strategies that aim to establish a vaccine-literate environment that can address limits in personal, community, and population-level VL. They also present an opportunity to enhance vaccine literacy as a whole.

Several stakeholders have the potential to play a crucial role in establishing an environment that promotes VL. This can be achieved through the development of easily accessible and straightforward information campaigns, fostering active community participation, and encouraging other entities to enhance their communication efforts. For example, nongovernmental organizations such as the World Health Organization and the United Nations International Children's Emergency Fund (UNICEF, now officially the United Nations Children's Fund), have been instrumental in promoting accurate information about vaccination. Additionally, specific donors have played a significant role in supporting vaccine literacy initiatives. Similarly, global business groups and their global and national affiliates such as the USCIB Foundation's Business partners to CONVINCE initiative, have developed educational modules to foster vaccine confidence and literacy [76]. Various stakeholders at the community level have undertaken impactful actions to enhance vaccine literacy, such as the New York Vaccine Literacy Campaign. These collective efforts hold the potential to contribute to the overall improvement of VL [77].

### Vaccine literacy and health literacy

The term HL appeared for the first time in the international literature in 1959; n 1974, Scott K. Simonds referred to HL as an outcome of health education [78, 79]. Approximately 20 years later, Parker [80] stated that “adequate functional health literacy means being able to apply literacy skills to health-related materials such as prescriptions, appointment cards, medicine labels, and direction for home care”. By the end of the last century and throughout the early 2000s, the concept of HL had already acquired many other meanings, with implications related to health care, disease prevention, and health promotion [10, 70]. Moreover, different levels or domains have been described, including functional, interactive, and critical domains [70]. With the growing interest and the international debate on HL, specific subareas were born. This was the case, for example, for VL, corona-specific HL and nutrition literacy [81, 82]. Most recently, HL has been conceptualized as a social vaccine in the context of the COVID-19 pandemic [8]. Although initially these subareas were merely considered as applications of the HL concept to specific health or health-related issues, the deepening of the debate both from both conceptual-theoretical and experimental points of view has increasingly led to understand that: *i.)* the concept of “general” HL and that of “specific” HL are partially – but not completely – overlap; *ii)* people with a high level of “general” HL do not necessarily also present a high level of “specific” HL; *iii)* “general” and “specific” HL tend to have a different weights in predicting some health or health-related outcomes. Therefore, HL and vaccine literacy should be considered only partially overlapping, because competencies and knowledge on vaccine, vaccination, and vaccination programms are very specific, and even people with a wide range of HL skills may be lacking in specific abilities that encompass vaccination, especially from the community point of view. In fact, a specific science, vaccinology, deals with the many aspects related to vaccines and vaccinations, not only biomedical and epidemiological, but also social, including health communication, economics, ethics, and politics. As previously mentioned, this is of particular relevance because vaccinations not only protect individuals but also contribute to enhancing population health. In fact, through the development of herd immunity, vaccinations play a crucial role in the elimination and eradication of many infectious diseases.

Additionally, vaccinations generally represent primary prevention tools devoted to healthy people, which at times may require an assumption of responsibility and decisions on behalf of others (e.g., parents with respect to their children), thus representing a peculiar and specific issue. Moreover, studies have reported that predictors of vaccination uptake or acceptance (e.g., educational levels and socioeconomic status) may differ from those of other health behaviours, thus suggesting that the personal reasons to get vaccinated may be different from reasons that determine people’s decision to adopt (or not) other preventive behaviours [83].

### Vaccine literacy and vaccine hesitancy

Vaccine hesitancy has been described in multiple ways. It is mostly addressed as "a behaviour of refusing or postponing vaccines despite their availability” [84, 85]. This definition focuses on the behavioural aspect of the construct, which may lead to labelling even nonvaccination due to communication issues or forgetfulness as hesitancy. In contrast, other authors place more emphasis on the cognitive aspect of vaccine hesitancy, defining it as “a position of uncertainty regarding the inoculation of a vaccine” [86, 87]. The most accepted and supported definition of vaccine hesitancy is given by the Strategic Advisory Group of Experts on Immunization (SAGE) Working Group dealing with vaccine hesitancy (2015):” Vaccine hesitancy refers to delay in acceptance or refusal of vaccines despite availability of vaccination services. Vaccine hesitancy is complex and context specific, varying across time, place, and vaccines. It is influenced by factors such as complacency, convenience, and confidence”, and basically integrates the two previous definitions [88].

According to the proposed definitions, personal, community and organizational VL share many aspects with the psychological determinants of vaccine hesitancy described in the “3C” model and in its evolutions (“4C”, “5C”, and the most recent “7C” model), although they remain distinct concepts. The 3C model was developed by the SAGE Working Group to map three main factors that influence vaccine uptake: confidence barriers, complacency barriers and convenience barriers [88]. In the following models (4C and 5C), other factors were added to better explain vaccine hesitancy (Table 2) [88–90]. All the models comprise several concepts from psychological theories, such as the Health Belief Model [91] and the Theory of Planned Behaviour [92, 93], to describe general attitudes towards vaccination and predicting prevention behaviour [89, 90]. During the COVID-19 pandemic, Geigere et al. [94] has introduced two other components, compliance with vaccination policies and conspiracy theory,to better understand the vaccination readiness, i.e., whether individuals are ready and willing to receive vaccinations (Table 2). Additionally, in the newest and expanded model (7C), the components refer to personal attitudes towards vaccination, from the personal and psychological perspectives. VL, as we have defined it in this manuscript, includes part of these components in the “motivation” factors, but also entails other aspects, such as knowledge and competencies, that are not included in the psychological determinants. In this sense, a vaccine-literate environment is defined by a wide range of abilities of the community, population, and organization, that affect motivation, similarto the “convenience” component of the models for vaccine hesitancy/acceptance, as well as knowledge and competencies.

**Table 2:** Components of the models for vaccine hesitancy or acceptance

Component	Definition	Model in which the component was introduced
Complacency [90]	Perceived risks if diseases are low; low involvement; vaccination not seen as necessary and as the injunctive norm	3C
Convenience [90]	Physical availability, affordability and willingness-to-pay, geographical accessibility, ability to understand	3C
Confidence [90]	Trust in effectiveness and safety of vaccines and the system that delivers them	3C
Calculation [91]	The degree to which personal costs and benefits of vaccination are weighted	4C
Collective responsibility [91]	The tendency to consider the protection of others in the decision to receive vaccines	5C
Compliance with vaccination policies [94]	Support for societal monitoring and sanctioning of people who are not vaccinated	7C
Conspiracy [94]	Conspiracy thinking and belief in fake news related to vaccination	7C

**Fig. 2:**
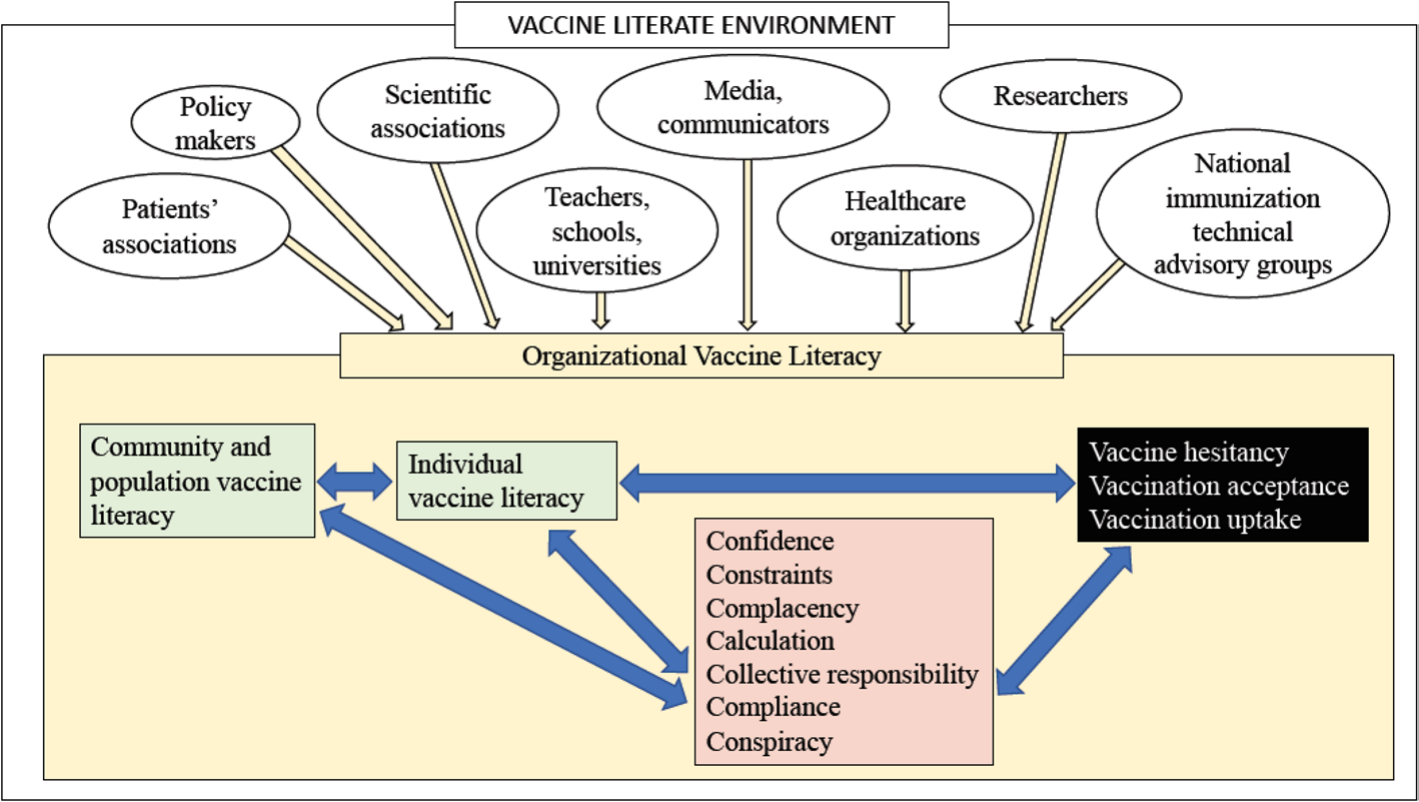
Personal, community, population, and organizational vaccine literacy, and vaccine hesitancy/acceptance

For all these reasons, VL can be considered as a set of competencies related to, but different from, the psychological determinants of vaccine hesitancy/acceptance (Fig. 2).

## Conclusions

The definitions of personal, community, population, and organizational VL proposed in this paper were developed by integrating and enriching existing definitions to outline the competencies, the actions, and the objectives to be considered either by policy-makers, or by researchers. From the perspective of policy-maker, the definitions and the description of the attributes of a VLHO provide a new paradigm to implement public health strategies that can help vaccination be understood as a social practice by the entire community. From the research perspective, our proposal defines the conceptual foundations, skills, and civic orientation to be taken into account when developing measurement tools devoted to assessing VL at different levels and in different contexts. Future studies that aim to deepen the relationship between VL, HL and the components of the models for vaccine hesitancy or acceptance are encouraged to shed further light on this complex link.

### Supplementary Information

**Additional file 1:** Supplementary Table 1: Characteristics and definitions of vaccine literacy of the studies included in the review.

## Data Availability

The datasets used and/or analysed during the current study available from the corresponding author on reasonable request.

## Abbreviations

HL: Health literacy
VL: Vaccine literacy
OVL: Organizational Vaccine Literacy
VLHO: Vaccine literate healthcare organization
OHL: Organizational Health Literacy
