# Correction: Vaccination as a social practice: towards a definition of personal, community, population, and organizational vaccine literacy

**DOI:** 10.1186/s12889-023-16550-6

**Published:** 2023-08-29

**Authors:** Chiara Lorini, Marco Del Riccio, Patrizio Zanobini, Luigi Roberto Biasio, Paolo Bonanni, Duccio Giorgetti, Valerio Ferro Allodola, Andrea Guazzini, Olfa Maghrebi, Vieri Lastrucci, Lisa Rigon, Orkan Okan, Kristine Sørensen, Guglielmo Bonaccorsi

**Affiliations:** 1 Department of Health Sciences, University of Florence, Viale Giovanni Battista Morgagni 48, 50134 Florence, Italy; 2 Health Literacy Laboratory (HeLiLab), University of Florence, Viale Giovanni Battista Morgagni 48, 50134 Florence, Italy; 3 Fondazione Giovanni Lorenzini, Viale Piave 35, 20129 Milan, Italy; 4 Medical School of Specialization in Hygiene and Preventive Medicine, University of Florence, Largo Giovanni Alessandro Brambilla 3, 50134 Florence, Italy; 5 Department of Law, Economics and Human Sciences, Mediterranea University of Reggio Calabria, Via Dell’Università 25, 89124 Reggio Calabria, Italy; 6 Department of Education, Literatures, Intercultural Studies, Languages and Psychology, University of Florence, Via Di San Salvi 12, 50135 Florence, Italy; 7 Epidemiology Unit, Meyer’s Children University Hospital, Viale Gaetano Pieraccini, 24, 50139 Florence, Italy; 8 Department of Sport and Health Sciences, Technical University of Munich, 80992 Munich, Germany; 9 Global Health Literacy Academy, Viengevej 100, 8240 Risskov, Denmark

**Correction: BMC Public Health 23, 1501 (2023)**

**https://doi.org/10.1186/s12889-023-16437-6**

The family and given names of the authors in the original publication were swapped. The correct information is shown in this correction article, the original article [1] has been updated.
