# Supplementary material for: Vaccination as a social practice: towards a definition of personal, community, population, and organizational vaccine literacy
Source: BMC Public Health. 2023 Aug 8;23:1501. doi: 10.1186/s12889-023-16437-6 (PMC10408168; doi:10.1186/s12889-023-16437-6)
Supplement: Supplementary file 1 — Additional file 1: Supplementary Table 1: Characteristics and definitions of vaccine literacy of the studies included in the review. [file 12889_2023_16437_MOESM1_ESM.docx]

**Supplementary Table 1**

| **First Author (year)** | **Country** | **Title** | **Definition of Vaccine Literacy** | **Levels of Vaccine Literacy** |
| --- | --- | --- | --- | --- |
| Adepoju P (2021) | Nigeria | Africa is waging a war on COVID anti-vaxxers | "[...] the right message and information are shared, and that the right education, what is called vaccine literacy, is actually promoted" | Personal |
| Adongo CA (2021) | Ghana | Beyond fragmentary: A proposed measure for travel vaccination concerns | "[Vaccine literacy concerns] inability to optimally obtain, process, understand and make an informed vaccination decision" | Personal |
| Arias A (2022) | France | Perception and Coverage of Conventional Vaccination among University Students from Rouen (Normandy), France in 2021 | it has been defined as a process of providing vaccine information, building communication, and increasing people’s engagement with vaccines | Organizational |
| Badua AR (2022) | Philippines | Vaccine literacy: A concept analysis | [...] a process of providing vaccine information, building communication, and increasing people’s engagement about vaccines. [...] vaccine literacy is connected with the motivation, knowledge, and competence of people about vaccines if they are capable of understanding and using this information to get vaccinate children and adults. | Personal, organizational |
| Biasio LR (2017) | Italy | Vaccine hesitancy and health literacy | "[Vaccine literacy] should not be considered simply knowledge about vaccines, but also developing a system with decreased complexity to communicate and offer vaccines" | Personal, organizational |
| Biasio LR (2019) | Italy | Vaccine literacy is undervalued | "[Vaccine literacy is] not simply knowledge about vaccines, but also developing a system with decreased complexity to communicate and offer vaccines as sine qua non of a functioning health system” | Personal |
| Biasio LR (2020) | Italy | Validation of an Italian tool to assess vaccine literacy in adulthood vaccination: a pilot study | "[Vaccine literacy] involves people's knowledge, motivation and competence to find, understand and use information to take decision about children's and adult's vaccination"; "[Vaccine literacy is] not simply knowledge about vaccines, but it entails motivation and competence to deal with information about immunization, disease prevention and also health promotion" | Personal, organizational |
| Biasio LR (2021) | Italy | Italian Adults' Likelihood of Getting COVID-19 Vaccine: A Second Online Survey | "[Vaccine literacy concerns] health literacy skills about vaccination"; "[Vaccine literacy concerns] individuals’ abilities to collect, understand, comprehend and use information about vaccines" | Personal |
| Biasio LR (2021) | Italy | Assessing COVID-19 vaccine literacy: a preliminary online survey | "[Vaccine literacy concerns] health literacy skills about vaccination [...] entailing people’s knowledge, motivation and competence to find, understand and use health information"; "[Vaccine literacy is] not simply knowledge about vaccines, but also developing a system with decreased complexity to communicate and offer vaccines as sine qua non of a functioning health system" | Personal, organizational |
| Bonaccorsi G (2019) | Italy | Non-Familial paid caregivers as potential flu carriers and cause of spread: the primary prevention of flu measured through their adhesion to flu vaccination campaigns—A Florentine experience | "[Vaccine literacy] is linked to literacy and entails people’s knowledge, motivation and competencies to access, understand, appraise, and apply health information in order to make judgments and take decisions in everyday life concerning healthcare, disease prevention and health promotion to maintain or improve quality of life during the life course" | Personal |
| Brieger D (2017) | Australia | Knowledge, attitudes and opinions towards measles and the MMR vaccine across two NSW cohorts | "Vaccine literacy can be approached as a variant of health literacy, which is defined [...] as “the knowledge, motivation and competencies of a consumer to access, understand, appraise and apply health information to make effective decisions about health and healthcare and take appropriate action” | Personal |
| Budiyanti RT (2021) | Indonesia | Barrier Factors Related to COVID-19 Vaccine Literacy in Developing Countries: A Traditional Literature Review | "COVID-19 vaccine literacy is the part of health literacy, which is knowledge, motivation, and competency to access, understand, and apply about COVID-19 vaccination and make a decision regarding COVID-19 vaccination to improve quality of life. This literacy is a balance between individual, community and also population skills in complexity system. Literacy skills regarding COVID-19 vaccination are fundamental for people to process data and experience related to vaccination programs, improve critical and systemic thinking, and decide whether to accept the vaccination or not" | Personal, organizational, population |
| Bullini Orlandi L (2022) | Italy | To do or not to do? Technological and social factors affecting vaccine coverage | "The VL concept embraces the same aspects of HL, such as “education—and knowledge about immunizations,” yet also entails “developing a system with decreased complexity to communicate and offer vaccines as sine qua non of a functioning health system” | Personal, organizational |
| Cadeddu C (2022) | Italy | The Determinants of Vaccine Literacy in the Italian Population: Results from the Health Literacy Survey 2019 | people’s knowledge, motivation and skills to find, understand and evaluate immunisation-related information in order to make adequate immunisation decisions | Personal |
| Carter J (2022) | Australia | Covid-19 vaccine uptake among younger women in rural Australia | "Functional vaccine literacy, defined as basic comprehension and interactive-critical vaccine literacy, defined as ability to critically analyze and apply meaning" | Personal |
| Correa-Rodríguez M (2022) | Spain | COVID-19 vaccine literacy in patients with systemic autoimmune diseases | not simply knowledge about vaccines, but also developing a system with decreased complexity to communicate and ofer vaccines as sine qua non of a functioning health system | Personal, organizational |
| Costantini H (2021) | Japan | COVID-19 Vaccine Literacy of Family Carers for Their Older Parents in Japan | "The notion of ‘vaccine literacy’, related to although distinct from health literacy, encompassing an understanding of the potential benefits from vaccination, the risks of side effects, as well as the economic costs and organizational process to access vaccination. In turn, vaccine literacy is contingent on personal circumstances as well as the broader societal context, thus contributing to shape intentions to vaccinate and ultimately vaccine uptake" | Personal, organizational, population |
| Engelbrecht MC (2022) | South Africa | COVID-19 Vaccine Hesitancy in South Africa: Lessons for Future Pandemics | Does not just denote knowledge about vaccines, but also refers to the (health) system’s ability to communicate clear and easyto-understand information about vaccines | Personal, organizational |
| Engelbrecht MC (2022) | South Africa | Factors Associated with Limited Vaccine Literacy: Lessons Learnt from COVID-19 | Vaccine literacy (VL) emanates from the concept of health literacy, which is defined as the “degree to which people have the capacity to obtain, process, and understand basic health information and services to make appropriate health decisions”. According to the same author, vaccine literacy does not just include knowledge about vaccines, but is also about “developing a system with decreased complexity to communicate and offer vaccines as sine qua non of a functioning health system”. | Personal, organizational |
| Fadda G (2022) | Italy | A survey about the degree of information and awareness of adolescents regarding vaccination in a Province of Central Italy | motivation and capability to obtain and understand information about vaccines from their parents or other sources (teachers, paediatricians or other doctors, vaccination centre, web, institutional sites, social networks, books, TV/radio); | Personal |
| Fadda M (2015) | Switzerland | Addressing issues of vaccination literacy and psychological empowerment in the measles-mumps-rubella (MMR) vaccination decision-making: a qualitative study | "vaccination literacy has to entail, among other skills such as factual knowledge on the risks and benefits of the vaccination, a correct understanding of the scope of current vaccination policies, since these parents questioned the need for vaccinating" | Personal |
| Fadda M (2017) | Switzerland | Beyond the knowledge gap paradigm: the role of psychological empowerment in parents’ vaccination decision | "Vaccination literacy can be seen as the context-specific counterpart of health literacy, defined as “the capacity to acquire, understand and use information in ways which promote and maintain good health” ; "Vaccination literacy can be conceptualized as a multi-dimensional construct comprising parents’ knowledge about vaccinations and their ability to find, judge and use vaccination-related information" | Personal |
| Ferris CA (2017) | Switzerland | Social Ecological Factors Associated with Parental Vaccination Decisions and Perceptions of Barriers to Childhood Immunizations | "The five defining attributes of parental vaccine literacy are: information access acquisition skills, understanding and comprehension skills, appraisal skills, application skills, and knowledge about childhood immunizations"; "Parental vaccine literacy is the ability to acquire information about vaccines, read, comprehend, and assess the credibility of that information, and use the resulting knowledge to make informed immunization decisions for one’s children." | Personal |
| Fry CA (2016) | USA | Addressing Pneumococcal Vaccine Uptake Disparities among African‐American Adults in the United States | "Health literacy and, more specifically, vaccine literacy, is the capacity of an individual to obtain, process, and understand vaccine information" | Personal |
| Gendler Y (2021) | Israel | Investigating the Influence of Vaccine Literacy, Vaccine Perception and Vaccine Hesitancy on Israeli Parents' Acceptance of the COVID-19 Vaccine for Their Children: A Cross-Sectional Study | "Vaccine literacy [is described] not only as a level of knowledge about vaccination, but also as a system with decreased complexity to communicate and offer vaccines as a necessity in a functioning health system" | Personal, organizational |
| Groenewald C (2022) | South Africa | To Vaccinate or Not? Decision-Making in the Time of COVID-19 Vaccines | "Vaccine literacy is thus not only about providing knowledge or creating awareness about the availability of vaccines but also includes the development of “a system with decreased complexity to communicate and offer vaccines as sine qua non of a functioning health system” | Personal, organizational |
| Gusar I (2021) | Croatia | Pre-vaccination COVID-19 vaccine literacy in a croatian adult population: A cross-sectional study | "The concept of vaccine literacy (VL) is founded on the concept of health literacy and is defined not only as a level of knowledge about vaccination but also as the development/construction of a system that would facilitate the communication or spread of messages about vaccines as being necessary, without which the functioning of the health system would be impossible" | Personal, organizational |
| Heiss SN (2015) | USA | Effects of interpersonal communication, knowledge, and attitudes on pertussis vaccination in Vermont | "Vaccine literacy is a complex concept because it involves understanding the health issue, the treatment options, appreciation of the larger global impact of vaccines, and being a critical consumer of anti-vaccine rhetoric" | Personal |
| Langford AT (2020) | USA | Health Communication and Decision Making about Vaccine Clinical Trials during a Pandemic | “Vaccine literacy” falls under the larger umbrella of health literacy and is defined as “not simply knowledge about vaccines, but also developing a system with decreased complexity to communicate and offer vaccines as sine qua non of a functioning health system” | Personal, organizational |
| Larson HJ (2020) | UK | Building Confidence to CONVINCE | "Vaccine literacy occurs when people understand, in their own language and relevant to their context, the content, processes and systems needed to access and get vaccinated. Vaccine literacy means knowing how and why vaccines work, the diseases they prevent, and their value to yourself and to society" | Personal |
| Li Y (2022) | China | The Development and Preliminary Application of the Chinese Version of the COVID-19 Vaccine Literacy Scale | Ratzan defined it as “not just knowledge about vaccines, it is closer to the development of a reduced complexity system that can be used by people to communicate and provide vaccine-related information, which is the premise and foundation of the stable and orderly operation of the health system. This system aims to strengthen the social norms of vaccination and provide a foundation of vaccine literacy appropriate to age, mind, gender, and environment for achieving herd immunity” [27]. Some scholars have pointed out that different from the concept of health literacy, vaccine literacy was more targeted for vaccination [28]. Vaccine literacy referred to “people’s knowledge, motivation and ability to discover, understand and use information to make vaccination decisions”. | Personal, organizational |
| Lorini C (2018) | Italy | Health literacy and vaccination: A systematic review | "Vaccine literacy is not simply knowledge about vaccines, but also developing a system with decreased complexity to communicate and offer vaccines as sine qua non of a functioning health system" | Personal, organizational |
| Lorini C (2020) | Italy | Health literacy, vaccine confidence and influenza vaccination uptake among nursing home staff: a cross-sectional study conducted in Tuscany | "[Vaccine literacy is] not simply knowledge about vaccines, but also developing a system with decreased complexity to communicate and offer vaccines as sine qua non of a functioning health system” | Personal, organizational |
| Lorini C (2022) | Italy | Vaccine Literacy and Source of Information about Vaccination among Staff of Nursing Homes: A Cross-Sectional Survey Conducted in Tuscany (Italy) | it aims to ensure that everyone understands what they need to know and do to get vaccinated and occurs when the skills and abilities of people align with the content, processes, and systems needed to access vaccines and get vaccinated. […] it can be considered as a tool mediating the transfer of information and facilitating vaccination acceptance, so communication about vaccination should be lined up with peoples’ VL | Personal |
| MacDonald N (2009) | Canada | Canada's eight-step vaccine safety program: Vaccine literacy | "[Vaccine literacy is] a better understanding of the many built-in safety monitoring components that ensure that vaccines are as safe as possible" | Personal |
| Maki W (2022) | Japan | Vaccine Literacy, COVID-19 Vaccine-Related Concerns, and Intention to Recommend COVID-19 Vaccines of Healthcare Workers in a Pediatric and Maternity Hospital: A Cross-Sectional Study | Vaccine literacy, which involves “not simply knowledge about vaccines, but also developing a system with decreased complexity to communicate and offer vaccines as sine qua non of a functioning health system | Personal, organizational |
| Maneesriwongul W (2022) | Thailand | Translation and Psychometric Testing of the Thai COVID-19 Vaccine Literacy Scale | "The VL [...] involves “people’s knowledge, motivation and competence to find, understand and use the information to make decisions about vaccination” | Personal |
| Masiello MM (2020) | USA | Building Vaccine Literacy in a Pandemic: How One Team of Public Health Students Is Responding | "Being ‘vaccine literate’ during the COVID-19 pandemic implies that what everyone needs to know and do to achieve population vaccination aligns with the public’s skills and abilities to find, use, and get the required vaccine (or vaccine series)"; "Vaccine literacy, a component of broader health literacy, is dependent on knowledge of the essential ‘need to know and do’ information, so in addition to learning how to identify and prioritize the most essential information" | Personal, population |
| Michel JP (2021) | Switzerland | Education, Healthy Ageing and Vaccine Literacy | "Health and vaccine literacy encompass people’s knowledge, motivation, and competence to access, understand, appraise and apply health information in order to make judgements and take decisions in everyday life concerning health care, disease prevention and health promotion"; "[Vaccine literacy] requires the individual to seek out relevant information among the ever-increasing data glut in the media (particularly on the Internet) and make an appropriate decision about vaccination" | Personal |
| Michel JP (2021) | Switzerland | Healthy Ageing and Vaccines: Application of the P4 Medicine Concept to Immunizations | "Vaccine literacy requires the individual to seek out relevant information among the ever-increasing data glut in the media (particularly on the Internet) and make an appropriate decision about vaccination" | Personal |
| Millar BC (2021) | UK | Improving vaccine-related health literacy in parents: comparison on the readability of CDC Vaccine Information Statements (VIS) and Health and Human Services (HHS) patient-facing vaccine literature | "[Vaccine literacy is] the ability to understand and act on health information" | Personal |
| Nkereuwem OO (2021) | Gambia | The use of a speaking book® to enhance vaccine knowledge among caregivers in The Gambia: A study using qualitative and quantitative methods | "Vaccine literacy is more complex than ‘just’ being knowledgeable about schedules and target diseases of the vaccines [..] it encompasses understanding, appraisal and application of vaccine-related information services" | Personal |
| Olson O (2020) | USA | Addressing parental vaccine hesitancy towards childhood vaccines in the united states: A systematic literature review of communication interventions and strategies | "Vaccine literacy is defined as not only knowledge about vaccines but the ability to use critical and evaluation skills to seek out the right information, especially with the ever-increasing information available in the media, particularly on the Internet" | Personal |
| Otieno NA (2020) | Kenya | Drivers and barriers of vaccine acceptance among pregnant women in Kenya | "[Vaccine literacy] is the degree to which a person has the capacity to obtain, process, and understand basic vaccine information and services to help them make appropriate health decision" | Personal |
| Popa AD (2022) | Romania | Determinants of the Hesitancy toward COVID-19 Vaccination in Eastern European Countries and the Relationship with Health and Vaccine Literacy: A Literature Review | it’s a degree of understanding the concept of vaccination and the establishment of a system that would assist the transmission or distribution of messages about the necessity of vaccination, without which a working health system would be impossible | Personal |
| Ratzan SC (2011) | USA | Vaccine Literacy, a Crucial Healthcare Innovation | “A new “Vaccine literacy” advocacy could be built on the idea of Health literacy, defined as the degree to which people have the capacity to obtain, process, and understand basic health information and services to make appropriate health decisions.” | Personal |
| Ratzan SC (2011) | USA | Vaccine literacy: a new shot for advancing health | ‘‘Vaccine literacy’’ advocacy could be built on the idea of ‘‘health literacy,’’ defined as the degree to which people have the capacity to obtain, process, and understand basic health information and services to make appropriate health decisions"; "Vaccine literacy is not simply knowledge about vaccines, but also developing a system with decreased complexity to communicate and offer vaccines as sine qua non of a functioning health system" | Personal, organizational |
| Ratzan SC (2020) | USA | Vaccine Literacy-helping Everyone Decide to Accept Vaccination | "Vaccine literacy occurs when the skills and abilities of people align with the content, processes, and systems needed to access and get vaccinated. Everyone, or their caretaker or family members, should be able to understand what they need to know and do related to their health, and specifically now for Covid-19 vaccination. Ultimately, this is dependent on content, processes, and systems for vaccination being easy to successfully access, navigate, and use" | Personal, organizational, population |
| Rauh (2020) | USA | A Select Bibliography of Actions to Promote Vaccine Literacy: A Resource for Health Communication | “Vaccine literacy is a fundamental health literacy urgently required to address this current pandemic. Vaccine literacy occurs when the skills and abilities of people align with the content, processes, and systems needed to access and get vaccinated. Everyone, or their caretaker or family members, should be able to understand what they need to know and do related to their health, and specifically now for vaccination for COVID. Ultimately, this is dependent on content, processes, and systems for vaccination being easy to successfully access, navigate, and use.” | Personal, organizational, population |
| Stockwell MS (2015) | USA | Text message reminders for second dose of influenza vaccine: a randomized controlled trial | "[Limited vaccine literacy] includes lack of knowledge and understanding about influenza and influenza vaccine and confusion regarding need for multiple doses and timing of doses" | Personal |
| Takahashi Y (2022) | Japan | COVID-19 vaccine literacy and vaccine hesitancy among pregnant women and mothers of young children in Japan | Vaccine literacy” (VL) is a critical determinant of vaccine uptake. VL is defined as ‘‘not simply knowledge about vaccines, but also developing a system with decreased complexity to communicate and offer vaccines as sine qua non of a functioning health system”. The concept of VL has been built upon the same characteristics as those of health literacy, that is, the cognitive and social skills that directly affect an individual’s ability to access and understand health information efficiently and make appropriate decisions | Personal, organizational |
| Yadete T (2021) | USA | Assessing acceptability of covid-19 vaccine booster dose among adult americans: A cross-sectional study | "Vaccine literacy is a person’s ability to collect and understand reliable information about immunizations and use the acquired knowledge to make informed decisions to benefit their health" | Personal |
| Yilmaz D (2022) | Turkey | Determining Covid-19 Vaccine Literacy Levels of Nursing Students | Vaccine literacy, which is one of the types of literacy, is indicated as one’s capacity to obtain, and understand basic health information and services in order to do suitable health decisions about vaccines  Vaccine literacy is also defined not only as having knowledge about vaccines but also as developing a system with less complexity to explain and present vaccines as an indispensable part of a functional health system | Personal, organizational |
